# Systematic Analysis of Endometrial Cancer-Associated Hub Proteins Based on Text Mining

**DOI:** 10.1155/2015/615825

**Published:** 2015-08-23

**Authors:** Huiqiao Gao, Zhenyu Zhang

**Affiliations:** Department of Obstetrics and Gynecology, Beijing Chao-yang Hospital, Capital Medical University, Beijing 100020, China

## Abstract

*Objective*. The aim of this study was to systematically characterize the expression of endometrial cancer- (EC-) associated genes and to analysis the functions, pathways, and networks of EC-associated hub proteins. *Methods*. Gene data for EC were extracted from the PubMed (MEDLINE) database using text mining based on NLP. PPI networks and pathways were integrated and obtained from the KEGG and other databases. Proteins that interacted with at least 10 other proteins were identified as the hub proteins of the EC-related genes network. *Results*. A total of 489 genes were identified as EC-related with *P* < 0.05, and 32 pathways were identified as significant (*P* < 0.05, FDR < 0.05). A network of EC-related proteins that included 271 interactions was constructed. The 17 proteins that interact with 10 or more other proteins (*P* < 0.05, FDR < 0.05) were identified as the hub proteins of this PPI network of EC-related genes. These 17 proteins are EGFR, MET, PDGFRB, CCND1, JUN, FGFR2, MYC, PIK3CA, PIK3R1, PIK3R2, KRAS, MAPK3, CTNNB1, RELA, JAK2, AKT1, and AKT2. *Conclusion*. Our data may help to reveal the molecular mechanisms of EC development and provide implications for targeted therapy for EC. However, corrections between certain proteins and EC continue to require additional exploration.

## 1. Introduction

Endometrial cancer is one of the most common gynecologic malignancies, and the incidence of this cancer continues to increase [1]. During the prior several decades, progress in molecular biology has improved our understanding of the occurrence and development of EC. It has been established that the biological behavior of tumors is controlled by functional proteins within cells and the signaling pathways in which these proteins are involved. Therefore, studies on the structure and function of hub proteins in signaling pathways may be valuable for diagnosing EC and for determining targeted therapies for this disease. To date, research has examined a large number of EC-related genes and proteins that could potentially be used as biomarkers or targets for diagnosis or treatment [2, 3]. However, most published papers regarding EC have focused on only a handful of genes and proteins. Although the research objectives of molecular biology are shifting from single genes or proteins to genomics or proteomics, there are a limited number of systematic studies of whole-genome expression in the context of EC.

At present, text mining (TM) technology is widely used in biomedical research to extract information from large quantities of biomedical literature and construct databases of disease-related genes, proteins, and molecular interactions [4, 5]. In this study, we systematically characterized the expression of EC-associated genes by mining data from the PubMed document retrieval system. In addition, we used bioinformatics methods to analyze the functions, pathways, and networks of relevant hub proteins.

## 2. Materials and Methods

The extraction of data by TM was based on natural language processing (NLP). Using “Endometrial Cancer” and “Endometrium Carcinoma” as search terms, we searched the PubMed database for article abstracts published before March 2014 and formatted the documents that were obtained. Genes and proteins that appeared in the abstracts of these documents were located and tagged using ABNER (A Biomedical Named Entity Recognizer; an open source tool for automatically tagging genes, proteins, and other entity names in text) [6]. Gene names were normalized based on the Entrez Gene database (the National Center for Biotechnology Information's database for gene-specific information) [7]. The frequency at which each gene occurred was then counted. A hypergeometric distribution was used to calculate the probabilities that genes would be cocited with EC at frequencies higher than theoretical expectations; genes of which *P* < 0.05 were considered relevant.

Gene ontology (GO) analysis was performed using GSEABase software package from the R statistical platform (http://www.r-project.org/). Genes were classified by biological process, cellular component, and molecular function. The EC-related protein-protein interaction (PPI) network was integrated from the KEGG (Kyoto Encyclopedia of Genes and Genomes), MIPS (Munich Information Center for Protein Sequences), and PubMed databases. GenMAPP v2.1 was used to map EC-related genes to the KEGG database to determine the pathways in which these genes were involved. A threshold of 0.05 was established for *P* values and false discovery rate (FDR).

## 3. Results

### 3.1. EC-Related Genes and GO Analysis

After the retrieval of documents from PubMed, 15157 abstracts were examined, and 832 genes were obtained. Eventually, a total of 489 genes were identified as EC-related with *P* < 0.05; among these genes, PGR, TP53, and MLH1 were mentioned most frequently.  Table 1 lists the 20 most significant EC-related genes.

Classification results for biological processes, cellular components, and molecular functions by GO analysis are presented in Table 2. Developmental processes, protein metabolism, and signal transduction were the major biological processes associated with EC-related genes; with respect to molecular function, the primary activities of these genes included signal transduction, nucleic acid binding, and transcriptional regulation. These genes were related to various cellular components, including the nucleus, plasma membrane, and nonstructural extracellular matrix.

### 3.2. Pathway and PPI Analysis

Following pathway analysis, 32 pathways were identified as significant (*P* < 0.05, FDR < 0.05); among these pathways, the cytokine-cytokine receptor interaction, MAPK, and focal adhesion signaling pathways involved the largest number of genes.  Table 3 lists the 20 most significant EC-related pathways.

**Figure 1:**
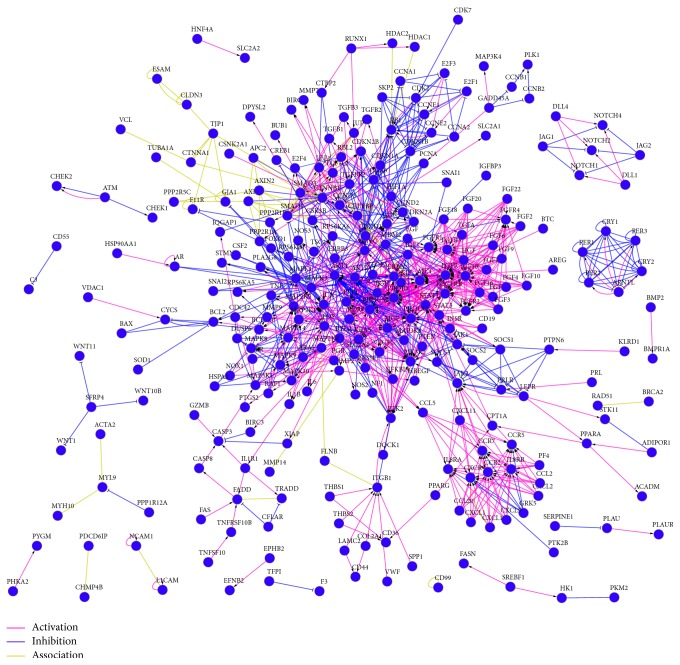
Network analysis of EC-related genes.

**Figure 2:**
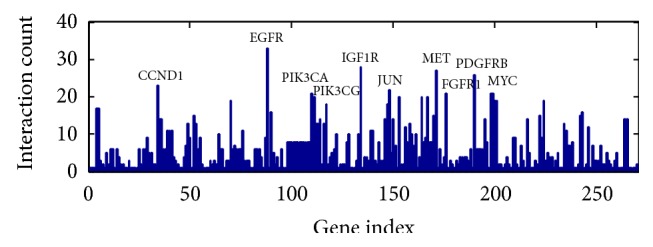
Hub proteins for EC.

We constructed a network of EC-related proteins that included 271 interactions (Figure 1). The 17 proteins that interact with at least 10 other proteins (*P* < 0.05,  FDR < 0.05) were identified as the hub proteins of the EC-related PPI network. These proteins are EGFR, MET, PDGFRB, CCND1, JUN, FGFR2, MYC, PIK3CA, PIK3R1, PIK3R2, KRAS, MAPK3, CTNNB1, RELA, JAK2, AKT1, and AKT2 (Figure 2). EGFR, which interacts with 33 other proteins, was the EC-related protein that exhibited the greatest number of interactions.

## 4. Discussion

In the present study, by extracting information from biomedical literature, we obtained a dataset of EC-related proteins and identified 17 hub proteins. Most relationships between EC and certain hub proteins, such as EGFR, IGF1R, and MET, have been extensively studied, and all of the aforementioned proteins are known to be closely related to the occurrence and development of EC. However, relative to these proteins, PDGFRB, FGFR2, MAPK3, and JAK2 have been reported less frequently in the context of EC.

### 4.1. PI3K and AKT

PI3K is a heterodimeric enzyme that consists of a regulatory subunit (p85) encoded by PIK3R1, PIK3R2, and PIK3R3 and a catalytic subunit (p110) encoded by PIK3CA, PIK3CB, and PIK3CD [8]. Mutations in PIK3CA, PIK3R1, and PIK3R2 occur at high rates in EC [9, 10]. AKT is the downstream target gene of PI3K, and AKT1 and AKT2 are two subtypes of AKT. Based on data mining, we found that PI3K and AKT are involved in many pathways, including the focal adhesion pathway, the toll-like receptor signaling pathway, and, most notably, the PI3K/AKT signaling pathway. PI3K phosphorylates PIP2 to PIP3, which can activate AKT. Subsequently, activated AKT stimulates the regulation of cellular metabolism, growth and survival by CCND1, Myc, NF-*κ*B, and a variety of downstream factors [11]. AKT plays a key role in this pathway. The PI3K/AKT signaling pathway can inhibit cell apoptosis and promote cell proliferation [12]. In EC, molecular alterations lead to increased PI3K/AKT signaling; in particular, the dominant activation event is the loss of the PTEN protein, which is a tumor suppressor that negatively affects the PI3K signaling pathway [11, 13]. Many recent studies have demonstrated that the PI3K/AKT pathway is activated in all types of EC and that this activation is associated with the aggressiveness of this disease [14, 15]. Recently, certain PI3K/AKT pathway inhibitors have been evaluated in preclinical or early clinical trials [16].

### 4.2. RAS and MAPK

RAS is an oncogene that serves as a central focus for many signal transduction pathways associated with a high percentage of human tumors. Activating mutations in KRAS can be observed in EC [17]. A recent analysis of EC signal transduction indicated that KRAS mutation is associated with elevated phosphorylation of MEK1/2, ERK1/2, and p38MAPK [9]. In fact, many studies have indicated that the RAS/MAPK pathway is frequently upregulated in EC [18, 19]. Moreover, KRAS also interacts with the PI3K pathway. Notably, KRAS-induced carcinogenesis can be inhibited when the interaction between RAS and the PI3K catalytic subunit P110*α* is blocked in vitro [20]. In this study, we found that KRAS and MAPK were involved in many signaling pathways, such as the MAPK signaling pathway, pathways involved in regulating the actin cytoskeleton, and the ErbB signaling pathway. As a hub of various pathways, MAPK regulates a cascade of downstream genes that participate in cell proliferation and differentiation, including Bcl-2, c-Myc, rock, and RSK2, among others.

### 4.3. FGFR2

FGFR2 is one type of fibroblast growth factor receptor and a member of the RTK family. RTKs are well known for their role in tumorigenesis [21]. In addition, it has been demonstrated that activating mutations in FGFR2 are associated with multiple types of tumors, including EC. By utilizing immunohistochemistry and PCR to examine FGFR2 expression and the presence of FGFR2 mutations in endometrial carcinoma, Gatius et al. determined that FGFR2 acted as an oncogene in EC and that FGFR2 expression was positively correlated with tumor stage and grade [22]. In our study, FGFR2 was mainly found to be involved in the MAPK signaling pathway and the regulation of the actin cytoskeleton. In fact, FGF signaling can activate several downstream pathways, including both the RAS-MAPK pathway and the PI3K-AKT pathway [23]. There has long been interest in FGFR inhibitors, and many studies have demonstrated that FGFR inhibition can block the progression of FGFR2-mutated EC [24, 25]. The targeting of FGFR2 is a possible treatment strategy for endometrial carcinoma.

### 4.4. PDGFRB

PDGF is a major mitogen that mediates the growth of fibroblasts, smooth muscle cells, and other cell. This protein also has significant effects on the angiogenesis of endothelial cells. PDGF exerts its biological effects by binding to its two receptors, *α*-receptor (PDGFRA) and *β*-receptor (PDGFRB), which are located on the cell membrane. These PDGF receptors are also members of the RTK family. In vivo and in vitro research have indicated that the excessive expression of PDGF and PDGFR can be detected in breast, pancreas, colorectal, and other tumors [26, 27]. Liegl et al. demonstrated that PDFGRB can be detected in the endothelial cells of endometrial stromal sarcomas [28]. PDGFR-mediated signaling contributes to tumor angiogenesis, and PDGF can upregulate the expression of VEGF, which also has angiogenic effects. Our TM indicated that PDGFRB participated as an upstream factor in cytokine-cytokine receptor interaction, the MAPK signaling pathway, focal adhesion, and the regulation of actin cytoskeleton. Moreover, the targeting of PDGFR to inhibit tumor cell signal transduction may play a crucial antitumor role [29, 30].

### 4.5. JAK2

JAK2, a member of the JAK family, is widely distributed in the cytoplasm. This protein is involved in signal transduction during hematopoiesis and in the immune system; in particular, JAK2 plays important roles in the production of red blood cells and the activation of immune cells. Research has demonstrated that JAK2 is associated with multiple tumors. The constitutive activation of JAK2 has been detected in many malignant solid tumors, such as colon cancer, head and neck cancer, leukemia, multiple myeloma, and other blood diseases [31–33]. Several JAK2 inhibitors are currently being evaluated in clinical trials in patients [34, 35]. JAK2 forms several signal transduction pathways in combination with multiple members of the STAT family; among these pathways, the JAK2-STAT3 pathway is particularly prominent. The JAK2-STAT3 signaling pathway, which mediates cell proliferation, differentiation, and apoptosis, is a focal point of the cellular signaling network and is closely associated with tumorigenesis [36]. However, there exists little research addressing the correlation between EC and JAK2-STAT3. The research of Liu et al. and Gao et al. indicated that the leptin can promote EC growth via activating the JAK2-STAT3 signal pathway in obese patient [37, 38]. In our study, JAK2 not only participates in the JAK-STAT pathway but also can activate the downstream PI3K-AKT pathway.

In summary, in this investigation, we systematically analyzed EC-related genes and identified certain hub proteins and their pathways and networks. This systematic study may help to reveal the molecular mechanisms of EC development. However, the study results were obtained based on TM, which only considered previously published literatures; thus, the correlations between certain proteins and EC require additional explorations. Moreover, our data also provide implications for targeted therapy for EC. After obtaining deeper insight into the EC-related signaling network, additional hub protein inhibitors with stronger specificities will be developed. Anyhow, multiple hub proteins-targeted drugs will have broad potential for tumor treatment.

## Figures and Tables

**Table 1:** The 20 most significant EC-related genes based on text mining.

Gene	Description	Count	*P* value
PGR	Progesterone receptor	323	0
TP53	Tumor protein p53	296	0
MLH1	mutL homolog 1	150	0
PTEN	Phosphatase and tensin homolog	130	0
MSH2	mutS homolog 2	112	0
VEGFA	Vascular endothelial growth factor A	82	0
ERBB2	erb-b2 receptor tyrosine kinase 2 (HER2)	77	0
MSH6	mutS homolog 6	75	0
EGFR	Epidermal growth factor receptor	68	0
MKI67	Antigen identified by monoclonal antibody Ki-67	66	4.80*E* − 09
BCL2	B-cell CLL/lymphoma 2	54	0
CCND1	Cyclin D1	53	1.02*E* − 08
ESR1	Estrogen receptor 1	48	0
TCEAL1	Transcription elongation factor A (SII)-like 1	47	0
CDKN2A	Cyclin-dependent kinase inhibitor 2A (p16)	39	0
CYP19A1	Cytochrome P450, family 19, subfamily A, polypeptide 1	39	0
INS	insulin	36	0
PTGS2	Prostaglandin-endoperoxide synthase 2 (COX2)	34	0
PMS2	Postmeiotic segregation increased 2	33	0
PCNA	Proliferating cell nuclear antigen	32	0

**Table 2:** Classification results for biological processes, cellular components, and molecular functions by GO analysis.

Term	Count	*P* value
Biological process		
Cell cycle and proliferation	224	4.05*E* − 11
Stress response	160	5.51*E* − 11
Developmental processes	336	8.54*E* − 11
RNA metabolism	188	0.00031
DNA metabolism	67	0
Protein metabolism	254	1.07*E* − 10
Other metabolic processes	229	2.58*E* − 10
Cell organization and biogenesis	178	7.72*E* − 11
Cell-cell signaling	44	8.21*E* − 08
Signal transduction	245	0.00089
Cell adhesion	51	0.00284
Death	141	2.77*E* − 11
Other biological processes	436	5.94*E* − 06

Molecular function		
Transcription regulatory activity	107	8.46*E* − 10
Signal transduction activity	240	3.60*E* − 05
Enzyme regulator activity	48	0.01638
Nucleic acid binding activity	194	2.53*E* − 07
Kinase activity	84	1.32*E* − 08
Other molecular function	744	2.86*E* − 07

Cellular component		
Extracellular matrix	34	1.42*E* − 05
Nonstructural extracellular	180	1.09*E* − 10
Cytosol	53	5.50*E* − 11
Nucleus	306	1.77*E* − 09
Plasma membrane	186	0.00014
Translational apparatus	22	0.00148
Other cellular component	446	9.02*E* − 08

**Table 3:** The 20 most significant pathways in which EC-related genes were involved.

Term	Count	*P* value
Cytokine-cytokine receptor interaction	64	1.95*E* − 09
MAPK signaling pathway	62	2.91*E* − 08
Focal adhesion	52	1.12*E* − 08
Cell cycle	48	8.71*E* − 15
Regulation of actin cytoskeleton	46	2.43*E* − 05
Jak-STAT signaling pathway	39	2.25*E* − 06
Toll-like receptor signaling pathway	36	3.72*E* − 10
Chemokine signaling pathway	36	0.00170
p53 signaling pathway	34	1.63*E* − 14
Apoptosis	33	3.75*E* − 10
T cell receptor signaling pathway	33	1.55*E* − 07
Insulin signaling pathway	33	3.00*E* − 05
ErbB signaling pathway	32	1.77*E* − 09
Wnt signaling pathway	32	6.43*E* − 04
Neurotrophin signaling pathway	31	3.51*E* − 05
Natural killer cell-mediated cytotoxicity	28	0.00168
Steroid hormone biosynthesis	26	9.99*E* − 13
Adherens junction	24	7.63*E* − 06
Fc epsilon RI signaling pathway	24	9.69*E* − 06
NOD-like receptor signaling pathway	23	4.62*E* − 07
